# Intracranial Subarachnoid Haemorrhage Caused by Cervical Spinal Dural Arteriovenous Fistulas: Case Report

**DOI:** 10.3389/fneur.2021.685332

**Published:** 2021-08-10

**Authors:** Junjie Zhao, Yagmur Esemen, Neil Rane, Ramesh Nair

**Affiliations:** ^1^Department of Neuroscience, Imperial College Healthcare National Health Service Trust, London, United Kingdom; ^2^Department of Radiology, Imperial College Healthcare National Health Service Trust, London, United Kingdom

**Keywords:** subarachnoid haemorrhage, spinal dural arteriovenous fistula, interventional neuroradiology, indocyanine green angiography, neurosurgery

## Abstract

Cervical spinal vascular abnormalities commonly present with progressive myelopathy as a result of venous congestion. They are not very prone to bleed and tend to be underdiagnosed due to their subtle clinical presentation. We came across a rare case of intracranial subarachnoid haemorrhage caused by cervical spinal dural fistula in the Imperial College Healthcare NHS Trust Hospitals/UK in June 2020. We diagnosed the patient under strict evidence base medicine guidance, which otherwise would have been missed. We discussed the case in several multidisciplinary team (MDT) meetings, and patient was treated under the joint care of the neurology and neurosurgical teams. Patient made a full recovery and discharged home with no neurological defects or complications. Here, we reported this case with all the evidence we gathered from our MDT discussion. We hope our experience would help improve the diagnosis and management protocol for future patients with a similar condition.

## Introduction

Although ruptured cerebral aneurysms cause most spontaneous subarachnoid haemorrhages (SAHs), they can also result from various rare conditions in about 5–10% of patients (1). Approximately 1% of reported intracranial SAHs are related to spinal pathologies, such as arteriovenous lesions (2).

Cervical arteriovenous fistulas (AVFs) are rare vascular malformations found in 1–2% of patients with spinal AVFs. Typically, AVF symptoms are associated with venous congestion, which can include subtle, slow-onset paraparesis (40%), back pain or radiculopathy (28%), and sphincter disturbances that are exacerbated by activity (3).

Intracranial SAHs caused by spine vascular abnormalities are extremely rare. In a literature search, only five studies were found published in the past decade (3–7).

Here, we discuss a case of intracranial SAHs caused by cervical AVF, focusing on how we came to the diagnosis under strict evidence guidance and treatment plans made under several multidisciplinary team (MDT) discussions.

## Case Presentation

A 40-year-old female attended the hospital with a brief episode of chest tightness radiating toward her back, immediately followed by severe headache, neck stiffness, nausea and vomiting, and photophobia. She was previously fit and well with no past medical history and no history of trauma. She was not on any anticoagulation therapy and had no significant family history of cancer or neurological disorder.

On examination, she demonstrated neck stiffness and occipital tenderness. There were no other positive findings in her initial neurological examinations. Her Glasgow Coma Scale (GCS) was 15, her mental state and speech were normal, her cranial nerves were unaffected and she had good power on all four limbs and a normal gait.

**Figure 1:**
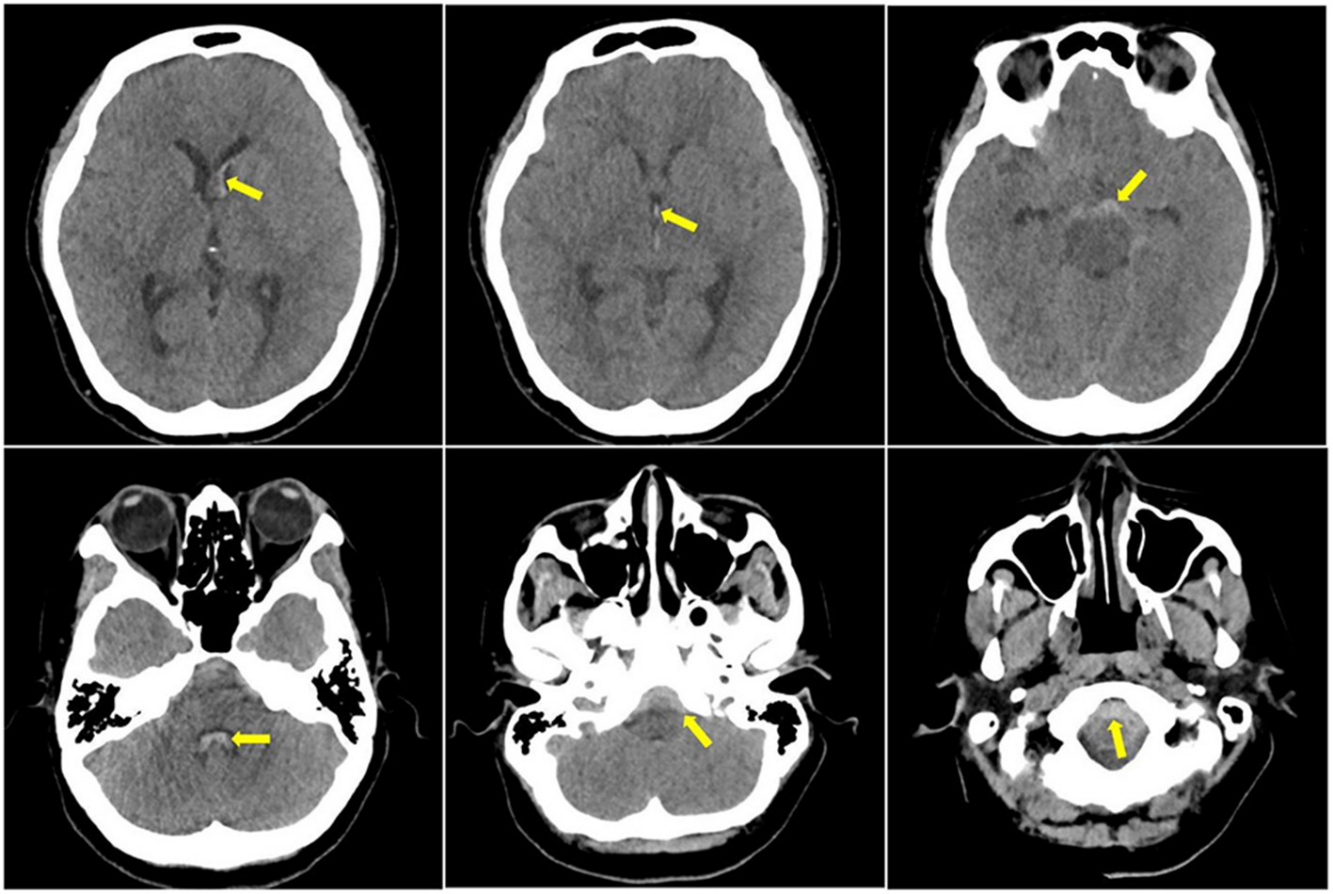
Initial CT head showed subarachnoid haemorrhage (SAH) present in the frontal horn of the left lateral ventricle, third ventricle and fourth ventricle, and the foramen magnum; yellow arrows indicate blood component in the scans.

A head CT was performed on arrival (level I evidence), which showed acute subarachnoid blood predominantly perimesencephalic in distribution with extension into the ventricular system (Figure 1). A CT angiography (CTA) intracranial and a cerebral digital subtraction angiography (DSA) was arranged within 24 h of admission (level I evidence) (8); both showed no convincing evidence of intracranial aneurysm or other vascular abnormalities.

**Figure 2:**
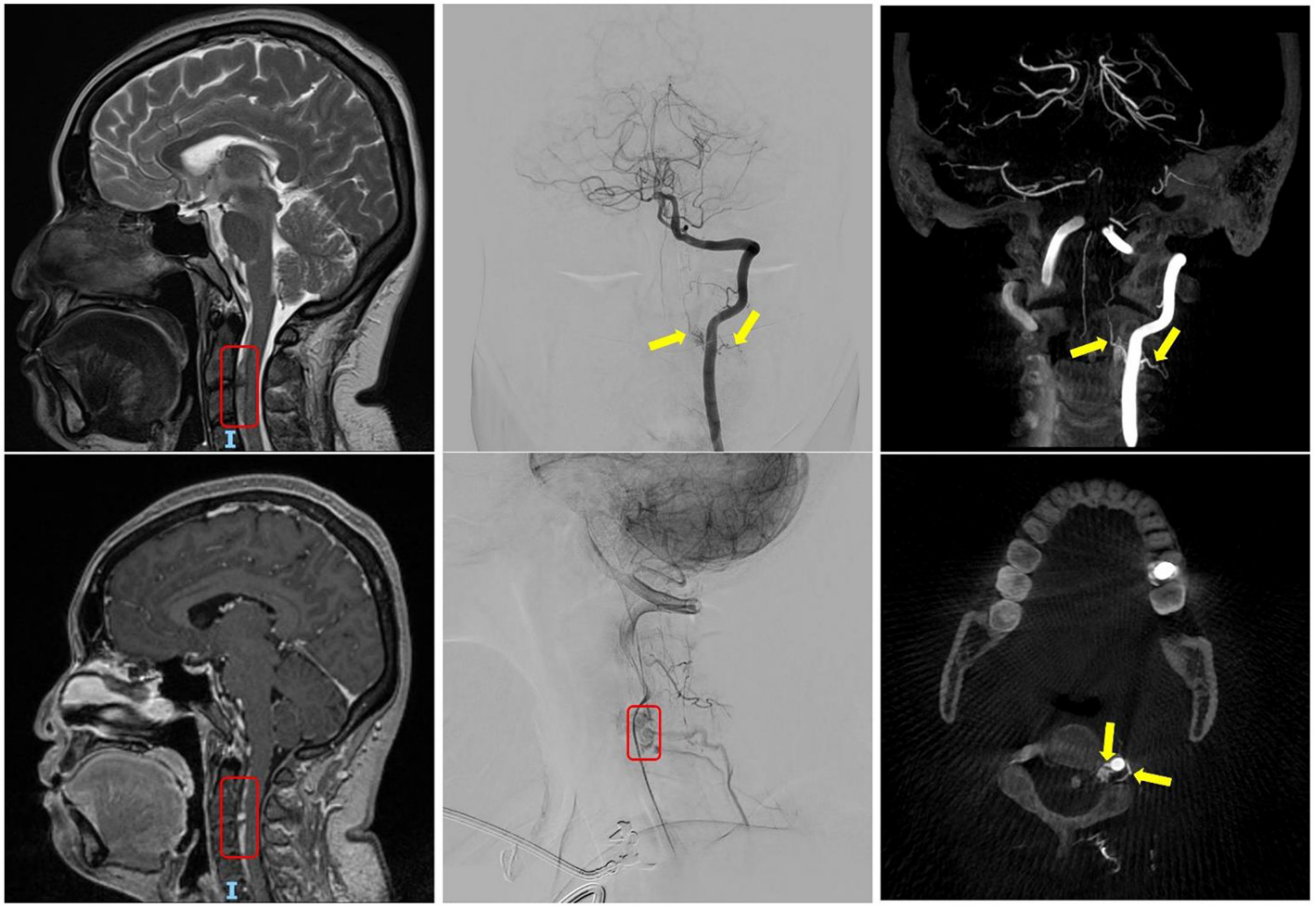
The left panels show the finding of the C2–3 dural arteriovenous fistula (AVF) in routine MRI head, highlighted within the red box. The middle and right panels show the digital subtraction angiography (DSA) cervical spine and the angio-CT of L-VA. The yellow arrows indicate the two supplying arteries; and the red boxes indicate tortuous venous drainage of the AVF.

The patient was subsequently closely managed under the joint care of neurology and neurosurgery. She was being monitored for signs of hydrocephalus and vasospasm during the acute stage (level I evidence). Her case was discussed in the first neuro-radiology MDT and was recommended for baseline MRI/MR angiography (MRA) 1 week into her SAH (level I evidence). The MRI/MRA result showed normal brain parenchyma and no evidence of an intracranial vascular malformation or aneurysm. However, there was a find of a small rounded lesion with enhancement at the level of C2–3, anterior to the cervical cord (Figure 2, left panels). This is consistent with an arteriovenous malformation (AVM)/AVF. A spinal DSA was organised, which showed a dural AVF situated at the ventral aspect of the cervical spine at the level of C2–3. Two radicular branches were coming off the left vertebral artery (VA) at the same level, travelling deep into the spinal canal and feed into the dural AVF. No anterior spinal artery (ASA) involvement was noted. It was a small 3–4-mm dilated varix in the midline and ventral to the cervical spinal cord, likely on the venous side, which is likely to represent the site of a recent haemorrhage (Figure 2, middle and right panels).

Her case was further discussed during the second neuroradiology MDT meeting, which included a neurologist, an interventional neuro-radiologist and neurosurgeons. Given that the two feeding branches are too small to navigate the micro-catheter, it was deemed unsuitable for endovascular treatment (level V evidence). Disconnection of dural AVF (level IV evidence) with intraoperative indocyanine green (ICG) angiogram guidance (level I evidence) was recommended by senior consultant neurosurgeons (9). The MDT decided to offer this patient surgery.

The patient fully consented and agreed to have surgery. Following surgery, she was neurologically well but complained of severe head and neck pain that had not improved since onset and very poor neck stiffness. Her pain was not controlled by regular paracetamol, codeine and morphine. Our pain team recommenced a ketamine trial (level I evidence), to which she responded well. Her headache dropped from a rating of 10/10–2/10 ~15 min after 25 mg of ketamine was administered. She subsequently commenced a 6-day course of ketamine 25 mg every 4 h for pain control, which was replaced with pregabalin 75 mg twice a day afterwards. Our physical therapist/occupational therapist (PT/OT) reviewed her neck stiffness and concluded that it was mainly due to subsequent fear of harming and avoiding activity/guarding of movement, which is fairly common in patients after spinal surgeries. They provided information for self-managing exercise and provided her with information on how to adapt movement to limit the pain and encourage a gradual return to normal activity (level II evidence). Other than physical symptoms, the patient had been very anxious during her postoperative recovery, mainly with the restrictions on inpatient management and no visitor rules due to COVID-19 restrictions. All patients found it difficult due to the lack of family support during this period. Our specialty nurse used iPads to arrange for family calls to ensure she has proper access to her loved ones, which helped calm her down and involve the family in her care.

With her treatment completed and symptoms fully controlled, she was discharged home from the hospital on the 10th postoperative day. The PT/OT had arranged for ongoing support from the community. Outpatient follow-up used virtual stream 6 weeks after discharge. The patient was well, was no longer using painkillers and had returned to her normal lifestyle.

**Figure 3:**
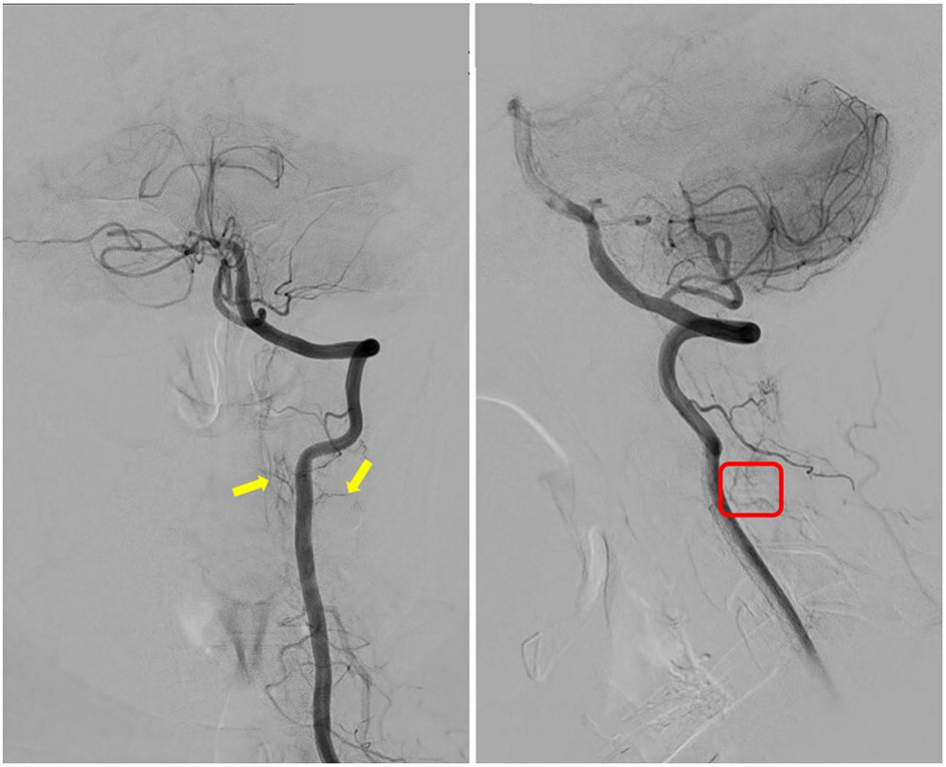
Follow-up spinal digital subtraction angiography (DSA) demonstrated images of L-VA; the yellow arrows indicate that the two supplying arteries no longer feed into the arteriovenous fistula (AVF); and red box indicates that tortuous venous drainage of the AVF had disappeared as compared with pre-op DSA image.

It was planned for her to receive a follow-up angio-spinal 6 months post operation per our neurovascular MDT recommendation. However, the procedure was delayed due to the COVID-19 restrictions. She eventually had follow-up spinal DSA 10 months post-surgery. The result showed that the C2–3 no longer demonstrated dural AVF, and no further arteriovenous shunting presented (Figure 3).

**Figure 4:**
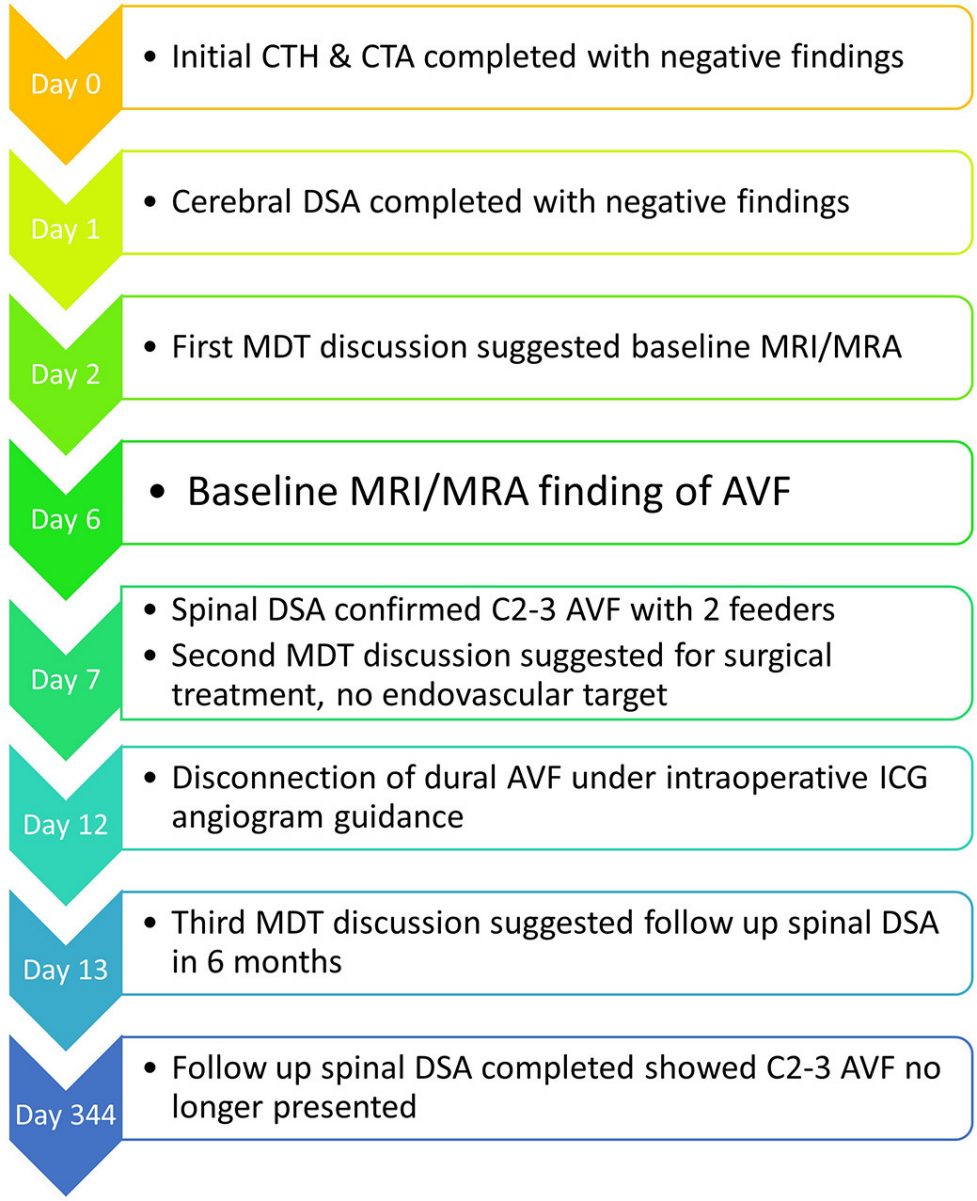
A diagram of timeline for this case.

Figure 4 summarises the timeline for this case.

## Discussion

### Spinal Dural Arteriovenous Fistulas: What Do We Know About Them?

A literature review suggested that, in most cases, spinal dural AVF presented with gradual onset myelopathy as venous drainage gradually fails. However, there are few documented cases of acute haemorrhages. They were all found by MRI and MRA of the whole spine when investigating non-aneurysmal SAH (NASAH). The mechanism by which the spinal bleeds cause the intracranial SAH is unclear. Typically, there is initially an intracranial SAH that then spread down to the spine. The result of intracranial SAH from the spinal lesion is most likely a reverse form of spreading from the typical scenario, consistent with blood pattern at foremen magnum. For intracranial AVMs, the Spetzler Martin Grading Scale was applied by evaluating AVM size, the pattern of venous drainage and eloquence of brain location to estimate the risk of surgery (10). However, for spinal AVM/AVFs, the classification system is not as straightforward. Historically, there had been three different systems of classifications.

The very first classification system was created between 1991 and 1998 by the combined efforts of different authors. The system describes four different types, as follows: type I, a single coiled vessel (spinal dural AVF); type II, intramedullary glomus AVM; type III, juvenile vessels; and type IV, intradural perimedullary (AVF). Different from the chronic presentation of most spinal AVMs or AVFs, type IV mostly presents acutely with more progressive myelopathy. Djindjian et al. (11) and Heros et al. (12) suggested further dividing type IV spinal AVF into three subtypes: subtype I, single arterial supply (ASA), single small fistula, slow ascending perimedullary venous drainage; subtype II, multiple arterial supplies [ASA and posterior spinal artery (PSA)], multiple medium fistulae, slow ascending perimedullary venous drainage; and subtype III, multiple arterial supplies (ASA and PSA), single giant fistula, large ectatic venous drainage.

Kim and Spetzler (13) in a review in 2006, proposed a modified classification system for spinal arteriovenous lesions based on specific anatomical and pathophysiological factors. The different types are described in the following: (1) extradural AVF, (2) intradural dorsal AVF, (3) intradural ventral AVF, (4) extra-intradural AVM, (5) intramedullary AVM, and (6) conus medullaris AVM.

Most recently in 2019, Lenck et al. (14) suggested the Toronto Classification of AVM/AVF of spine based on the anatomic feature and the topography of the shunting site. Under this system, the spinal vascular lesions were divided into the following: (a) spinal cord AVM—glomus intramedullary lesions; (b) pial AVF—shunts located superficial to the cord in the subpial space; (c) dural AVF—lesion located intradural but extrapial; (d) epidural AVF—lesions located outside dura but within the spinal canal; (e) paraspinal AVF—lesion pushing through to the outside of spinal canal and drain into para spinal venous plexuses; and (f) spinal arteriovenous metameric syndrome (SAMS)—lesions that involve multiple tissue layers (e.g., spinal cord, bone, paraspinal musculature, subcutaneous tissues, and skin) in one or several metameric segments.

Comparing among the three systems, our case here fitted mostly under the Toronto Classification as a type c dural AVF, as it had two radicular branches from L-VA that entered the dura and supply into a large ectatic venous drainage at the C2–3 level.

**Figure 5:**
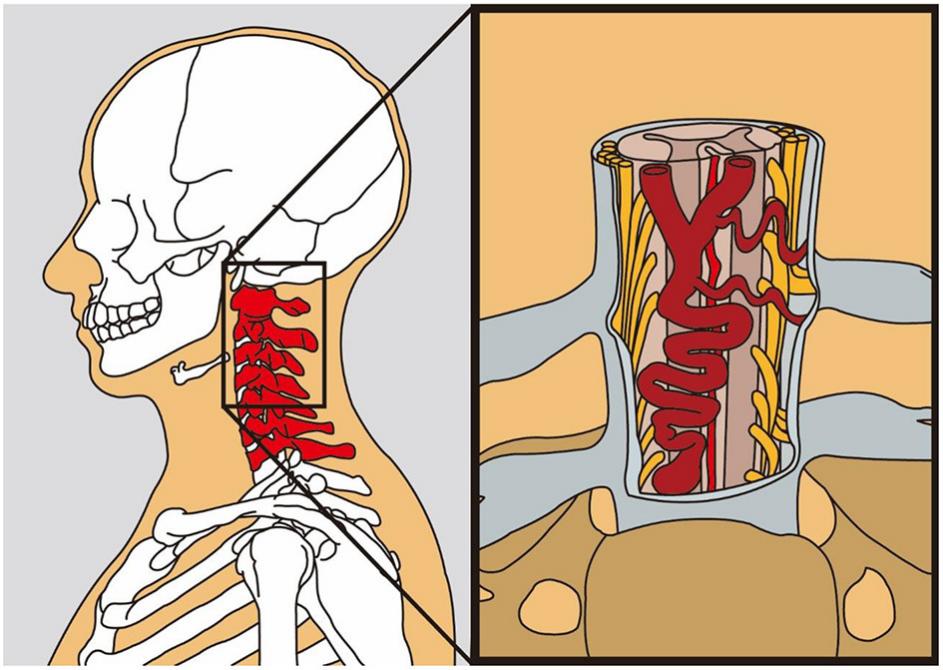
The schematic model of the dural arteriovenous fistula (AVF) lying in the ventral aspect of the cervical spinal cord (this figure is an artwork produced by Mr. Huang for us; he had kindly permitted us to publish his work in our manuscript).

A schematic image of the lesion is shown in Figure 5.

### Diagnosis Pathway: Routine Spinal Imagining?

Up to 10% of all spontaneous SAH are NASAHs. The evaluation of SAH patients with negative DSA is sometimes a diagnostic challenge. Kashefiolasl et al. studied 103 NASAH patients. Among them, 23 (22%) patients had a CT negative SAH, diagnosed by positive LP. All patients received an MRI of the spine; only two (1.9%) patients presented with a positive finding. Both patients complained of radicular sciatic pain. The detection rate increased by up to 25% in cases where patients with radicular sciatic pain at the presentation of symptoms received an MRI. The study concluded that routine radiological investigation of the spine in every NASAH patient is not recommended due to the rarity of the pathology and should be done as symptom orientated (7). Similar findings were also noted in five other studies investigating NASAH *via* MRI spine to locate the source of bleeding; they report only a 1% positive finding (15).

In our case, the patient was initially being treated as a NASAH, and the lesion was picked up during the routine follow-up MRI/MRA. If the lesion level was two vertebrae lower, the diagnosis of such a bleeding lesion would not have been found. In our literature research, within the past decade, there had been one case study describing intracranial SAH caused by a thoracic spinal vascular lesion (15). It was discussed with our neuro-radiologist if routine spinal imaging would be warranted for other NASAHs. It was concluded that given the history in our case that she did have one episode of chest tightness, radiating toward her back immediately prior to the headache, a full spine MRI was clinically warranted even if the baseline MRI/MRA head and neck was negative (level IV evidence). However, running routine spinal scans for all NASAH is still not recommended due to the rarity of the condition.

### Treatment Options

Limited resources are reporting the treatment options of spinal AVMs/AVFs. Hiramatsu et al. (3) reported the treatment outcome from a 59-patient cohort. Of the 59 lesions, 28 (47%) were treated with direct surgery only, and eight (14%) were managed conservatively. Fifteen lesions (25%) were treated with endovascular embolisation only. Eight lesions (14%) were treated with direct surgery and endovascular embolisation (3, 16–18). Unfortunately, no publications have presented and discussed follow-ups and the treatment outcome for those cohorts.

Following a discussion in our neurovascular MDT, the patient's lesion was deemed unsuitable for endovascular embolisation (level V evidence). The final decision was to offer her C2–3 posterior laminectomy and disconnection of dural AVF (level IV evidence). She was made aware of her condition, the rationale for this treatment option, and the risks and benefits associated with it. She made the informed decision of proceeding with the treatment.

## Conclusion

We diagnosed and treated a rare case of intracranial SAH secondary to cervical dural AVF guided by evidence-based medicine. Intracranial SAH caused by cervical spine vascular abnormalities is rare; however, in the event of such a case, it is essential to look for any spinal symptoms in NASAH. A full spinal MRI is clinically warranted for any NASAH patients presenting with transient or persistent spinal symptoms (level IV evidence).

Our patient recovered well after surgery under the joint care of our specialist nursing staff, neurologists, neurosurgeons, pain team, and rehabilitation therapists. The management of her case had again emphasised that collaboration of MDT is critical for patient recovery and could affect the overall experience for the patient (level I evidence).

## Data Availability Statement

The original contributions presented in the study are included in the article/Supplementary Material, further inquiries can be directed to the corresponding author/s.

## Ethics Statement

Ethical review and approval was not required for the study on human participants in accordance with the local legislation and institutional requirements. The patients/participants provided their written informed consent to participate in this study. Written informed consent was obtained from the individual(s) for the publication of any potentially identifiable images or data included in this article.

## Author Contributions

JZ performed the neurological examination, wrote the first draft of the article and was responsible for the overall content. NR evaluated the radiological images and contributed mainly to the discussion of the cases. RN performed the surgical treatment on the patient and contributed significantly to the patient's discussion and revision of the first draft. All authors reviewed and approved the final manuscript and ensured that all the questions regarding the accuracy of the article were appropriately investigated and resolved.

## Conflict of Interest

The authors declare that the research was conducted in the absence of any commercial or financial relationships that could be construed as a potential conflict of interest.

## Publisher's Note

All claims expressed in this article are solely those of the authors and do not necessarily represent those of their affiliated organizations, or those of the publisher, the editors and the reviewers. Any product that may be evaluated in this article, or claim that may be made by its manufacturer, is not guaranteed or endorsed by the publisher.
