# Supplementary material for: Intracranial Subarachnoid Haemorrhage Caused by Cervical Spinal Dural Arteriovenous Fistulas: Case Report
Source: Front Neurol. 2021 Aug 10;12:685332. doi: 10.3389/fneur.2021.685332 (PMC8383207; doi:10.3389/fneur.2021.685332)
Supplement: Supplementary file 1 [file Data_Sheet_1.docx]

# Supplementary Material

Surgical detail provided by neurosurgeons

C2-3 posterior laminectomy and disconnection of dural AVF were planned and performed by senior consultant neurosurgeons (level IV evidence). C2-3 level was confirmed with intraoperative imaging (II) before incision and exposure, followed by C2-3 laminectomy performed under microscopy. Spinal dura opened via midline and exposed carefully from the left side to ventral. Two tortuous arterial feed can be seen near the C3 nerve root travelling towards the spinal cord. The Dentate Ligament was divided to gently mobilize the spinal cord to expose the ventral aspect showing the tortuous vessels. The Indocyanine green (ICG) angiogram was done to confirm the flow through the tortuous vessels to the spinal cord. Bipolar coagulation to disconnect the two tortuous vessels from their communication to the dural AVF. Successful disconnection was confirmed with ICG injection that there was no flow of any further tortuous vessels connected to the spinal cord’s ventral aspect (level I evidence).

Intraoperative images have been displayed in figure A below:


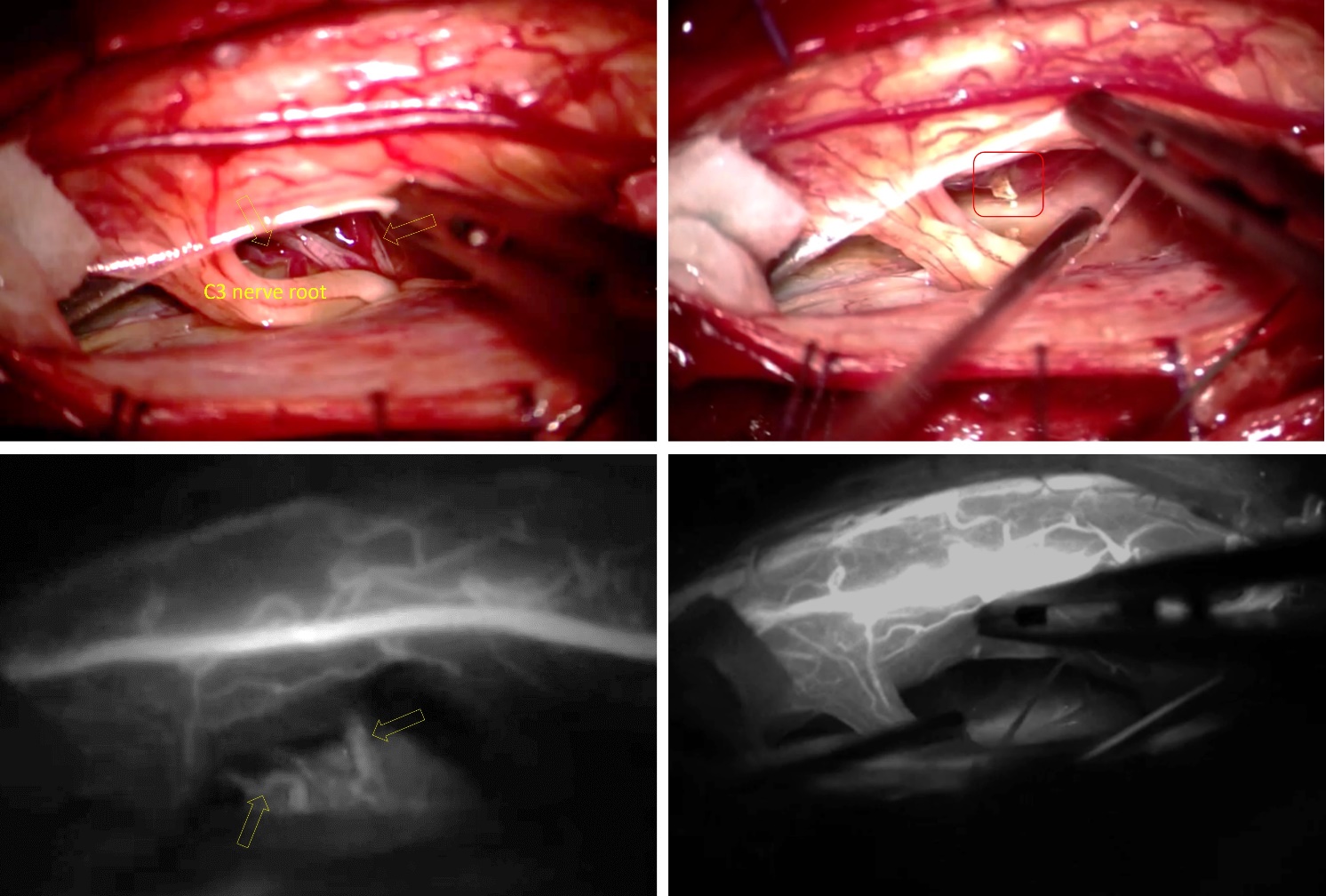


Figure A: Intraoperative images: The upper microscopic images show the AVF. The bottom images show the corresponding fluoroscopic AVF images post IVG injection. Yellow arrows indicating the two supplying arteries, and the red box indicated one of the terminals of the two arteries after being disconnected. The bottom left image shows the exposure showed 2 tortuous feeding arteries below the C3 nerve route supplying the AVF and one enlarged draining vein in between. The bottom right image shows R- post-disconnection of the 2-feeding artery.

Feedback from the patient’s perspective

“I’ve been through hell. This was the most traumatic experience I ever had in my life. For me, the pain is the most unbearable part of my entire experience, and I can still remember every moment vividly. I couldn’t sleep, which makes me feel so much worse. And I couldn’t do anything with the pain. The worst moment was during the angiogram (DSA), every time they inject the dye was like that fire burning from the side of my neck and the fire was ascending up to the tip of my head, adding to the pain I already had…

And it was because the pain was so bad that when the pain team came and offer me the ketamine trial, I felt for the first-time some moments of pain relief. And with that everything started to change for better. With that I managed to get some sleep and rest, and I manage to do more with therapists as well. Therefore, I really am thankful for the pain team’s effort.

Overall, this has been a positive experience for me despite all the suffering I’ve been through. The clinical team had kept me well informed of every next step in my treatment plan, it does help me a lot with my anxiety and it had lessened my frustration and stress under all that pain. All the nursing staff were amazing too, they had been extremely patient with me when I was in a very bad shape under all that pain. Also, the therapists are great too, the exercises they taught me to practice myself really help improve my neck stiffness.

The one thing I felt a bit of a shame was my families couldn’t visit me during my stay due to the covid situation.

However, the nursing staff offered me an iPad to video my families and they help passing-on a lot of personal items from my families to me, which make my heart warm and really help me cope with being isolated from my families and with all the pain I suffered from during my stay in hospital.”
